# Five-Year Survival after Surgery for Invasive Micropapillary Carcinoma of the Stomach

**DOI:** 10.1155/2013/560712

**Published:** 2013-03-27

**Authors:** Shigeo Ninomiya, Kazuya Sonoda, Hidefumi Shiroshita, Toshio Bandoh, Tsuyoshi Arita

**Affiliations:** Department of Surgery, Arita Gastrointestinal Hospital, 1-2-6 Maki, Oita 870-0294, Japan

## Abstract

Invasive micropapillary carcinoma (IMPC) of the breast, urinary bladder, ovary, and colon has been reported. However, few reports have described IMPC of the stomach. In addition, IMPC has been described as a histological indicator for lymphatic invasion and nodal metastasis, resulting in poor prognosis. We report a case of 5-year survival after surgery for IPMC of the stomach. A 69-year-old woman was admitted to our hospital with symptoms of upper abdominal pain. Upper gastrointestinal endoscopy revealed a tumor at the antrum of the stomach. Histological examination of the biopsy specimen indicated poorly differentiated adenocarcinoma. The patient underwent distal gastrectomy with lymph node dissection. Microscopic examination of the specimen revealed that the tumor consisted of an invasive micropapillary component. Carcinoma cell clusters were floating in the clear spaces. The patient recovered uneventfully and remains alive without recurrence 5 years after surgery.

## 1. Introduction

Invasive micropapillary carcinoma (IMPC) was first reported as a rare subtype of invasive ductal carcinoma of the breast, defined as a carcinoma composed of small clusters of tumor cells lying within clear spaces simulating vascular channels [1]. IMPC has been described as a histological indicator for lymphatic invasion and nodal metastasis, resulting in poor prognosis [2]. After initially being reported as a histological variant of breast cancer [1], IPMC of the urinary bladder [3], ovary [4], colon [5], and ampulla of Vater [6] was also reported. However, reports of IPMC of the stomach have been limited. Herein, we report a case of 5-year survival after surgery for IMPC of the stomach and discuss the clinical findings of this disease according to the previous literature.

## 2. Case Report

**Figure 1:**
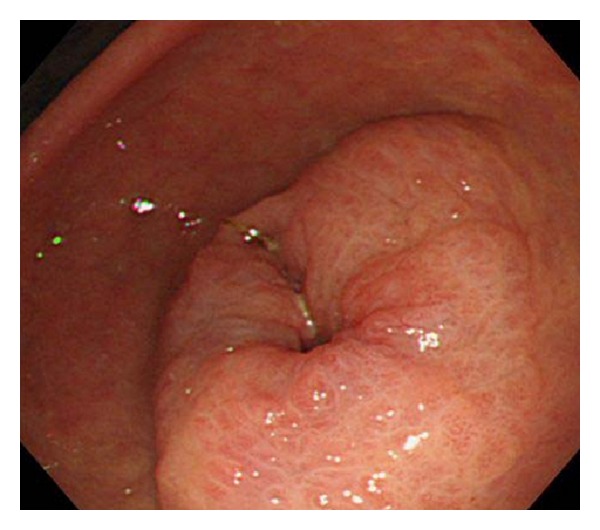
Upper gastrointestinal endoscopy revealed a circumferential tumor located at the antrum of the stomach.

**Figure 2:**
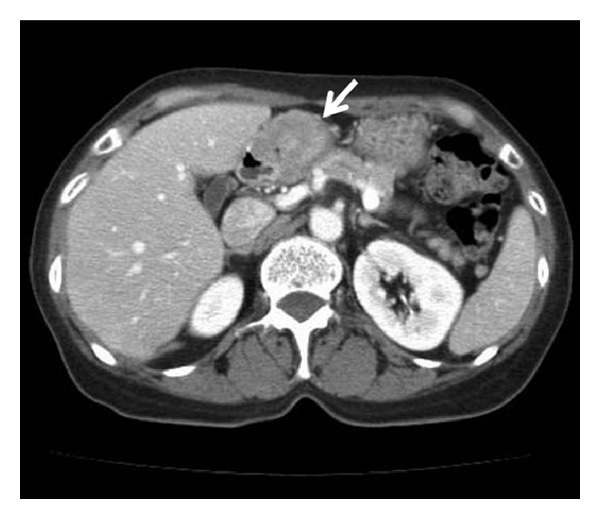
Computed tomography showed a tumor located at the antrum of the stomach (arrow), with no invasion of the primary tumor to adjacent structures and no distant metastasis.

**Figure 3:**
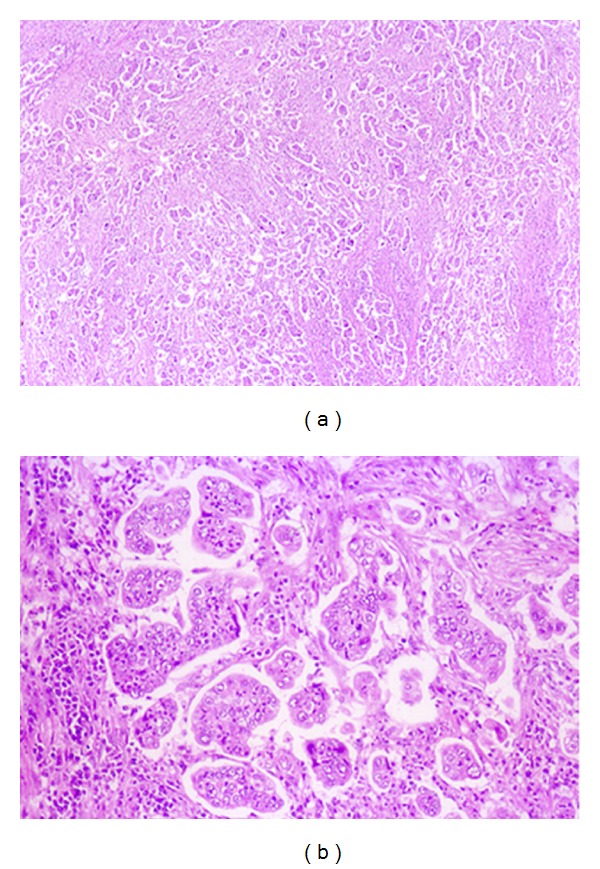
Histological examination of the specimen revealed that the tumor consisted of an invasive micropapillary component. Carcinoma cell clusters were floating in the clear spaces. (a) Hematoxylin-eosin stain, original magnification, ×40. (b) Hematoxylin-eosin stain, ×200.

**Figure 4:**
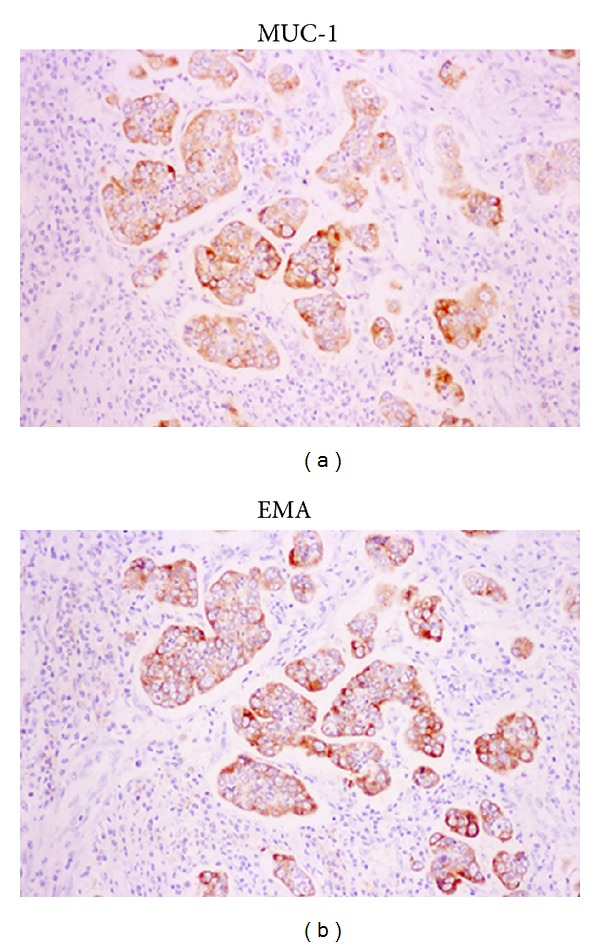
Immunohistochemical staining showed the carcinoma cells to be positive for epithelial membrane antigen (EMA) and MUC-1. Carcinoma cell membranes at the periphery of the cell clusters were characteristically positive for EMA and MUC-1, indicating an “inside-out” pattern of reactivity.

A 69-year-old woman was admitted to our hospital with the complaint of upper abdominal pain. On admission, her abdomen was flat and soft, and no superficial lymph nodes were palpated. Routine laboratory findings were unremarkable, with the exception of an elevated carbohydrate antigen 19-9 level of 544 U/mL. Upper gastrointestinal endoscopy revealed a circumferential tumor located at the antrum of the stomach (Figure 1). Histopathological examination indicated poorly differentiated adenocarcinoma. Computed tomography (CT) also showed a tumor located at the antrum of the stomach, with no invasion of the primary tumor to the adjacent structures and no distant metastasis (Figure 2). Based on all of the preoperative clinical examinations, the tumor was diagnosed to be T3N1M0 stage IIIA according to UICC staging. With a provisional diagnosis of gastric adenocarcinoma, we performed distal gastrectomy with lymph node dissection. Macroscopically, the tumor was 36 × 30 mm in size. Microscopic examination of the specimen revealed that the tumor consisted of an invasive micropapillary component. Carcinoma cell clusters were floating in the clear spaces (Figures 3(a) and 3(b)). Carcinoma cells had invaded the subserous tissue, and two regional lymph node metastases were seen. In addition, there was marked lymphatic invasion, but no obvious venous invasion. The sections were examined immunohistochemically with primary antibodies using the streptavidin peroxidase complex method, which revealed carcinoma cells positive for epithelial membrane antigen (EMA) and MUC-1. Also, carcinoma cell membranes at the periphery of the cell clusters were characteristically positive for EMA, indicating an “inside-out” pattern [7] of reactivity (Figures 4(a) and 4(b)).

We administered postoperative adjuvant chemotherapy with S-1 according to the pathological results. Presently, the patient is alive and without recurrence and is doing well 5 years after surgery.

## 3. Discussion

IMPC was initially described as a histological variant of breast cancer, characterized as a carcinoma composed of small clusters of tumor cells lying within clear spaces simulating vascular channels. IPMC of the breast has a high propensity to invade the lymphatic system with extensive metastasis to the axillary lymph nodes [1]. Although IPMC of the stomach is extremely rare, and this entity has not been fully described, the pathological features of IPMC of the stomach have been reported recently. Ushiku et al. [7] identified an incidence of nodal metastasis in 82% of cases, and lymphovascular invasion was present in all 17 cases. Fujita et al. [8] also reported an incidence of nodal metastasis of 100% and lymphatic invasion of 78.5% in their 14-case series. Therefore, the clinicopathological features of IPMC of the stomach could be similar to those of IPMC of the breast, with a high incidence of nodal metastasis and lymphatic invasion.

 The prognosis for patients with IPMC of the stomach has not been clarified. Some reports have shown survival rates no poorer than those of patients with IPMC of the breast. Fujita et al. [8] noted that significant differences were observed in the 3-year disease-free survival rate of patients with IPMC of the stomach compared with that for the stage-matched controls. Eom et al. [9] reported that overall 5-year survival rates for patients with IPMC of the stomach were significantly worse than those for patients with stage I and II non-IPMC. However, Roh et al. [10] found no significant differences in overall survival rates between patients with IPMC of the stomach and non-IPMC. These previous reports have some limitations in that the numbers of patients with IMPC of the stomach were very small. Our case is a rare report of 5-year survival from IPMC of the stomach successfully treated by surgery and adjuvant chemotherapy. Further examination is necessary to clarify the prognosis for patients with IPMC of the stomach.

 Optimal treatment strategy for IPMC of the stomach has not been established. This rare histological type frequently shows aggressive tumor behavior with marked lymphatic invasion and nodal metastasis in various organs. With recent chemotherapeutic advances, it is expected that adjuvant chemotherapy would result in a survival benefit after surgery for stages II and III gastric cancer [11]. Eom et al. [9] reported that IPMC of the stomach was predictive of worse patient survival rate especially in patients with stages I and II cancer. Adjuvant chemotherapy should be administered for the patients with IPMC of the stomach at high risk for recurrence. Additionally, a few reported cases of early-stage IPMC of the stomach exist in the literature [7, 10]. With the technical advance of endoscopic submucosal dissection (ESD), most early-stage gastric cancer could be treated without surgery. However, it is important not to hesitate to carry out surgery when pathological examination of the specimen after ESD reveals early-stage gastric cancer with an invasive micropapillary component.

## 4. Conclusion

We reported a case of 5-year survival after surgery for IPMC of the stomach. To clarify the features of IPMC of the stomach, including responses to chemotherapy, further investigations with a large number of cases are required.
